# Short communication: genomic kinship, opposing homozygotes and genetic diversity in a selected population of Australian Angus cattle

**DOI:** 10.1093/jas/skaf207

**Published:** 2025-06-19

**Authors:** Antonio Reverter, Malshani Samaraweera, Pâmela A Alexandre, Christian Duff, Laercio Porto-Neto

**Affiliations:** CSIRO Agriculture and Food, Brisbane, QLD, Australia; Angus Australia, Armidale, NSW, Australia; CSIRO Agriculture and Food, Brisbane, QLD, Australia; Angus Australia, Armidale, NSW, Australia; CSIRO Agriculture and Food, Brisbane, QLD, Australia

**Keywords:** beef cattle, angus, pedigree, genotype data, Mendelian inconsistencies, genetic diversity

## Abstract

Using molecular genotypes to check for Mendelian inconsistencies allows the identification of animals for which pedigree and genotype information disagree. A further use of molecular data is to understand and manage genetic diversity in a population. We sourced from the Angus Australia database a selected population of 11,224 animals, including 10,309 progeny born between 2013 and 2023 from 269 sires and 646 dams with at least 100 and 10 progeny, respectively. All animals had imputed genotypes for 61,105 autosomal markers. The additive and dominance genomic relationship (**GR**), as well as the number of opposing homozygotes (OH) were examined for pedigree-based relationship pairs including parent–offspring (PO; 21,307 pairs), full-sibs (**FS**; 35,486), half-sibs (**HS**; 677,421), grandparent–grandoffspring (16,308) and unrelated (62,232,954 pairs). Theoretical expectations for means and variances were compared against empirical observations. Consistent with expectations, the variance of additive GR among FS pairs was higher than the variance among HS, and the number of OH among FS was half the number of OH among HS. Expected to be 0.5, the observed additive GR among FS pairs and PO pairs was 0.483 (SD = 0.054) and 0.488 (SD = 0.037), respectively. The correlation (± SE) between additive and dominance GR was near unity for self-relationships (*r* = 0.935 ± 0.003) and zero for unrelated pairs (*r* = −0.001 ± 0.000). Expected to be zero, the number of OH among PO pairs averaged 11.6 and 77.5% of all PO pairs had an OH ≤ 12. Among FS pairs, the observed OH averaged 1,162.45 (expected = 1,150.17), and this average was surpassed by only 14 PO pairs, which was attributed to pedigree errors. Crucially, the anticipated negative correlation between additive GR and OH was affected by the degree of kindship being strongest negative among unrelated pairs (*r* = −0.762 ± 0.001). A principal components analysis and a network-based pipeline revealed the genetic diversity of the population with a focus on the role of the most influential parents. We conclude that, in our selected population of Australian Angus cattle, observed GRs were close to expectations, while Mendelian inconsistencies were very rare and likely attributed to either errors in pedigree recording, mislabeling of samples, or error in genotypes and genotype imputation. Finally, our study reveals the genetic diversity and breeding management decisions occurring in modern Australian Angus breeding programs.

## Teaser

By utilizing a specific group of Australian Angus cattle, we demonstrate that the comparison of pedigree and molecular data serves as an effective method for testing inheritance theory, understanding genetic diversity and identifying animal pairs where pedigree and genotype information may not align.

## Introduction

In livestock populations and over the last 3 decades, DNA technology has been hailed as the most accurate way to determine an animal’s parentage (Davis and DeNise, 1998). Indeed, the availability of genome-wide single nucleotide polymorphisms (**SNP**) genotypes enables the accurate estimation of the observed relationship between any individual pairs, not just PO. At the same time, beef cattle breed societies worldwide have a charter to maintain and archive a herd book of their registered cattle that includes pedigree details such as sire, dam, date of birth and sex. Often pedigree information traces back many generations which allows the estimation of the expected relationship between individual pairs.

A way to monitor and increase the accuracy and quality of pedigree information is through DNA testing and paternity verification. Parentage testing not only ensures correct pedigree recording but can provide information to make important management decisions for commercial producers. Knowing the most likely sire of each calf allows producers to make more informed decisions relative to a future grouping of bull cohorts based on calf performance, including potential calving difficulties. For instance, based on a large, commercial cattle ranch setting, Van Eenennaam et al. (2007) used DNA tests to assign paternity to calves from a multiple-sire breeding system. The authors reported a large variability in calf output and a large proportion of young bulls that did not sire any offspring. Additionally, producers using AI followed by natural service mating may have some calves with birth dates that are too ambiguous to determine the sire, and the only way to determine this correctly is by using parentage testing (Peters et al. 2013). This knowledge aids commercial producers who wish to retain only AI sired heifers as replacements or seedstock producers who rely on accurate pedigree information for breeding value predictions.

However, on any large, registered breeding population undergoing selection and spanning several generations, there is bound to be many animal pair relationships equivalent to parent–offspring based on additive genetic relationship alone. Therefore, the objective of this study was to source the herd book register (HBR) from Angus Australia (https://www.angusaustralia.com.au/) and to examine genetic diversity and the myriad of animal pair relationships that exists in a selected population of genotyped animals from highly used bulls and cows. A further objective was to use imputed genotypes for 61,105 autosomal SNP to explore the genetic diversity and invoke inheritance theory to check for Mendelian inconsistencies where pedigree and genotype information do not align. The assessment of Mendelian inconsistencies was conducted by examining additive genetic relationships, dominance genetic relationships, and counting opposing homozygotes (OH) where a given pair of animals exhibit homozygosity for different alleles at a particular locus.

## Materials and Methods

Animal Care and Use Committee approval was not obtained for this study because historical data were used, and no animals were handled as part of this study.

### Resource population and pedigree-based relationships

We accessed the herd book registry from Angus Australia to capture genotyped individuals born from 2013 to 2023 and from frequently used genotyped parents. The final population included 11,224 animals including 10,309 progeny from 269 sires with at least 100 progeny, and 646 dams with at least 10 progeny.

The number of animals born from years 2013 to 2023 (number of sires and dams in brackets) was respectively: 9 (6 sires and 6 dams), 24 (15, 21), 255 (34, 87), 505 (50,146), 718 (78, 207), 1,070 (100, 294), 1,557 (122, 366), 1,645 (131, 398), 2,242 (132, 410), 2,222 (122, 346), and 152 (28, 38). On average, the 269 sires had progeny in 3.04 yr, but ranges went from 1 yr (57 sires) to 10 yr (1 sire). Similarly, the 646 dams had on average progeny in 3.59 yr, but ranged from 1 yr (40 dams) to 9 yr (4 dams).

Pedigree information of the calves was recorded at registration, and DNA parentage testing conducted once they are genotyped. Therefore, for some animals, pedigree errors were identified and corrected prior to this study. However, only pedigree information was used to select the animals and stablish animal pairs included in this study. Tracing back 3 generations of pedigree information, this resource population contained the following 10 relationship pairs: (1) 11,224 self-relationships; (2) 35,486 full-sib (**FS**) pairs; (3) 10,892 sire–offspring pairs; (4) 10,415 dam–offspring pairs; (5) 584,845 paternal half-sibs (**HS**); (6) 92,576 maternal HS; (7) 8,059 paternal grandparent–grandprogeny; (8) 8,215 maternal grandparent–grandprogeny; (9) 34 paternal and maternal grandparent–grand progeny; and (1) 62,232,954 unknown relationships.

### Genomic-based relationships

All animals had imputed genotypes for 61,105 autosomal SNP used in the routine genomic evaluation of Angus Australia. Details of the imputation process are given in Aliloo and Clark (2021). In brief, the average imputation accuracy, calculated across 10-fold cross-validation, ranged from 0.97 to 0.99. Also, SNP with consistently low imputation accuracies across most chip panels were eliminated to ensure they are not used in imputation.

The panel of 61,105 SNP employed in this study contains 408 of the 554 recommended by the International Committee for Animal Recording (ICAR) for parentage verification. Based on the ARS_UCD1.2 bovine genomic assembly (Rosen et al., 2020), the number of SNP mapped to bovine autosomal chromosome 1 to 29 was respectively: 3,736, 3,384, 3,209, 2,997, 2,784, 2,970, 2,824, 2,743, 2,664, 2,439, 2,467, 1,798, 1,938, 2,118, 1,899, 1,834, 1,851, 1,611, 1,583, 1,991, 1,998, 1,622, 1,537, 1,393, 1,156, 1,232, 1,234, 984, and 1,109.

Among the 11,224 animals of the resource population and their 62,994,700 pairs, 3 types of genomic-based relationships were considered as follows:

(1) Additive genomic relationship (AGR): constructed following the method described by VanRaden (2008) :

$$\begin{array}{l} AGR=\frac{Z_{a}Z_{a}^{\prime }}{2\overset{m}{\underset{k=1}{\sum }}p_{k}q_{k}} \end{array}$$

where matrix ***Z***_*a*_ has dimensions of the number of individuals (*n*) by the number of loci (*m*), with elements that are equal to *2*−*2pk* and −*2pk* for opposite homozygous and *1*−*2pk* for heterozygous genotypes, *pk* is the minor allele frequency of locus *k*, and *qk = 1-pk*.

(2) Dominance genomic relationship (DGR): constructed following the method described by Vitezica et al. (2013):

$$\begin{array}{l} DGR=\frac{Z_{d}Z_{d}^{\prime }}{4\overset{m}{\underset{k=1}{\sum }}p_{k}^{2}q_{k}^{2}} \end{array}$$

where matrix ***Z***_*d*_ has dimensions of the number of individuals (*n*) by the number of loci (*m*), with elements that are equal to $-2q_{k}^{2}$ for genotype A_1_A_1_, 2p_k_q_k_ for genotype A_1_A_2_ and $-2p_{k}^{2}$ for genotype A_2_A_2_.

(3) Opposing homozygotes (OH): Two animals have OH loci when 1 animal is homozygous for 1 allele, and the other animal is homozygous for the other allele. The total number of OH instances was the sum up across all 61,105 SNP.

### Theoretical expectations

For all animal pairs, the observed distribution of additive and dominance GR, and that of OH values were compared with the expected distribution based on pedigree information and number of SNP and allele frequencies. Theoretical expectations for additive and dominance relationships were sourced from Lagervall (1960), Willham (1963) and Visscher (2009); while expected distributions for OH were derived from Calus et al. (2011) and Komiya et al. (2021).

### The special case of parents–offspring trios

Parents–offspring (PO) trios were given a special consideration because the interrelationship between parents governs the expected inbreeding in offspring, as well as PO relationships. Therefore, to ascertain the accuracy of the pedigree information and the quality of the genotype data, the observed additive GR for all parents–offspring trios in the resource population were compared against the theoretical expectations.

Following derivations from Grashei et al. (2018) who developed a GR likelihood method for the fast and accurate trio parentage assignment, the expected additive GR of an offspring (O) with its true parents (TP), P1 and P2 are


$$\begin{array}{l} E\left(AGR_{O,P1}|TP\right)=0.5\left(AGR_{P1,P1}+AGR_{P1,P2}\right) \end{array}$$

$$\begin{array}{l} E\left(AGR_{O,P2}|TP\right)=0.5\left(AGR_{P2,P2}+AGR_{P1,P2}\right) \end{array}$$

While the expected relationship of the offspring with itself is


$$\begin{array}{l} E\left(AGR_{O,O}|TP\right)=1+\;\;\;0.5AGR_{P1,P2} \end{array}$$

where $0.5AGR_{P1,P2}$ is the expected inbreeding coefficient of the offspring.

### Cluster analyses and genetic diversity from influential parents

To further understand genetic diversity, principal component analysis (**PCA**) was performed on the additive GR matrix to visualize potential clustering and identify those animals more distant from the majority. Following examples from other studies (e.g., Mudadu et al. (2016) and Steyn et al. (2022)), the animals falling within the extreme ends of the first 2 principal components were considered as key parents contributing to genetic variation within our selected population.

The genetic diversity of the resource population was examined after subjecting the top 10 sires and dams with the greatest number of progeny to the Pedigromics pipeline (Reverter et al. 2019) where the PO relationships are represented in a network diagram with features including animal type (e.g., progeny, sire, dam), year of birth and inbreeding mapped to node shape, color and size, respectively.

## Results and Discussion

Table 1 presents the expected and observed mean and SD values for additive and dominance GR, as well as the number of OH sites across the 10 pedigree-based relationships examined in this study. For the same set of metrics, ranges are given in Tables S1 and S2. After collapsing maternal and paternal HS, and maternal and paternal grandparents into single categories, Fig. S1 shows the empirical density distribution for additive GR across 6 distinct pedigree-based kinship categories.

**Table 1.** Expected (EXP) and observed (OBS) mean and SD values for additive and dominance GR, number of OH sites across 10 pedigree-based relationships examined in this study

Relationship	*N* Pairs	Additive GR			Dominance GR			OH		
		EXP	OBS	SD	EXP	OBS	SD	EXP	OBS	SD
Self	11,224	1.000	0.995	0.038	1.000	0.997	0.036	0	0	0
Full sib	35,486	0.500	0.483	0.054	0.250	0.239	0.052	1,150.57	1,162.45	286.811
Sire–offspring	10,892	0.500	0.485	0.036	0.000	−0.004	0.030	0	7.61	89.555
Dam–offspring	10,415	0.500	0.492	0.037	0.000	−0.004	0.030	0	15.74	122.403
Paternal half sib	584,845	0.250	0.239	0.044	0.000	0.005	0.027	2,300.34	2,268.40	352.754
Maternal half sib	92,576	0.250	0.240	0.048	0.000	0.002	0.030	2,300.34	2,262.03	362.211
Paternal grandparent	8,059	0.250	0.225	0.050	0.000	−0.003	0.021	2,300.34	2,316.99	417.580
Maternal grandparent	8,215	0.250	0.228	0.053	0.000	−0.005	0.022	2,300.34	2,298.27	419.669
Paternal and maternal grandparent	34	0.500	0.469	0.066	0.125	0.109	0.057	1,150.57	1,245.68	476.408
None	62,232,954	0.000	−0.003	0.038	0.000	0.000	0.012	4,600.67	4,507.88	449.325

Full-sib and PO pairs were expected to have the same additive GR of 0.5. However, the variance of additive GR for FS pairs was higher than that observed for PO pairs (SD = 0.054 vs. 0.037). The lower and upper bounds of the empirical 99% confidence interval for additive GR were respectively 0.349 and 0.623 for FS pairs, and 0.408 and 0.592 for PO pairs. This result was expected given that, in the absence of inbreeding, the genetic identity derived by PO descent cannot exceed 0.5 because the offspring inherits exactly one-half of the autosomal genes from each parent. To be precise, the relationship for the proportion of shared alleles between a parent and an offspring is exact, while the relationship between sibs is probabilistic.

Although HS pairs and grandparent–grandoffspring pairs have the same additive GR expectation of 0.25, the additive GR variance was larger for grandparent–grandoffspring pairs (SD = 0.053 vs. 0.045). Again, these results agree with expectations derived by Visscher (2009) showing that for non-collateral (i.e., case of ancestor-descendant) relatives, the more distant the relatives (and so the smaller the coefficient of relationship), the larger the SD of sharing as a proportion of the mean. For cattle populations with 30 chromosomes and genome map length of 30 Morgans, Hill (1993) estimated an SD of 0.035 for grandparent–grandoffspring pairs, and an SD of 0.028 for HS pairs. More recently, working with a multibreed population of purebred and crossbred cattle, Kenny et al. (2023) reported a SD for additive GR among FS pairs of 0.040 units which is closer to the 0.054 found here (Table 1). In a population of Texel sheep, Kaseja et al. (2022) reported a SD for additive GR among 238 FS pairs and among 4,994 PO pairs of 0.04 and 0.05, respectively. Our results are also very similar to those from Moore et al. (2019) with a population of Limousine cattle who reported a SD for additive GR among 83 FS pairs and among 3,167 PO pairs of 0.06 and 0.03, respectively.

Consistent with expectations, dominance GR was on average zero for all animal pairs except for self-relationships (observed 0.997, expected 1.000), FS (observed 0.239, expected 0.250) and the 34 instances of paternal and maternal grandparent–grandoffspring (observed 0.109, expected 0.125). While FS share dominance variation, offspring do not share dominance variation with their parents, only additive genetic variance. A closer examination of the 34 instances of paternal and maternal grandparent–grandoffspring pairs reveal these corresponded to inbred animals sharing the same paternal and maternal grandsire, or the same paternal and maternal granddam, and 2 resulting from the mating of FS which carries an expected PO additive and dominance relationship of 0.750 and 0.250, respectively.

The correlation between additive and dominance GR was at its highest and near unity for self-relationships (*r* = 0.935 ± 0.003), followed by FS pairs (*r* = 0.786 ± 0.003), PO pairs (*r* = 0.575 ± 0.008), HS pairs (*r* = 0.420 ± 0.001), grandparent–grandoffspring pairs (*r* = 0.328 ± 0.007), and down to zero for unrelated pairs (*r* = −0.001 ± 0.000). Fig. S2 illustrates the relationship between additive and dominance GR for 3 pedigree-based kinship categories: HS pairs, FS pairs and self-relationships. For non-inbred populations, Visscher (2009) estimated a correlation of 0.894 between the additive and dominance coefficients of relatedness in FS. The lower correlations observed in our dataset were attributed to the presence of inbreeding. For instance, for 883 animals found to have an estimated genomic inbreeding > 5%, the correlation between additive and dominance GR was 0.786 ± 0.021.

Clearly apparent from Fig. S2, there are potential pedigree errors for animal pairs registered as HS or FS and yet an estimated near zero additive and dominance GR. Similarly, Fig. S2 shows the presence of 4 FS pairs with a near unity additive and dominance GR. These were attributed to either 1 of 3 possibilities: 1) identical monozygotic twins (when born on the same date); or 2) the result of a cloned embryo frozen for later use (when born of different dates); or 3) the same animal registered and sampled twice with 2 different identifiers (also when born on different dates).

Expected to be zero, the observed number of OH was on average 7.61 (or 0.01% of SNP) for sire–offspring pairs and 15.74 (or 0.03% of SNP) for dam–offspring pairs (Table 1). The higher number of OH observed for dam–offspring pairs could be explained to having many more dams (646) than sires (269) increasing the random chance of pedigree errors on the dam side. Importantly, the distribution of OH values for PO pairs was heavily skewed with 79.4% of sire–offspring pairs having an OH ≤ 8, and 75.2% of dam–offspring pairs having an OH ≤ 16. Across all 21,307 PO pairs, the observed 95th and 99th percentiles for OH values were 34 and 62, respectively. Similarly, only 5 sire–offspring pairs (out of 10,892) and 9 dam–offspring pairs (out of 10.415) exhibited an OH > 1,150 (or 1.88% of SNP), which is the expectation for FS pairs with the same expected additive GR of 0.5 as PO pairs.

Previous studies have suggested the use of a nominal percentage of SNP as an exclusion based OH threshold for paternity testing (e.g., Calus et al. 2011; Strucken et al. 2016; Komiya et al. 2021). In particular, Strucken et al. (2016) reported that decreasing the threshold increased false negatives, and increasing it increased false positives, proposing 1% as most suitable threshold. In our dataset, taking a nominal 1% threshold (or 611 SNP) a total of 10,886 sire–offspring pairs (or 99.9%) and a total of 10,405 dam–offspring pairs (also 99.9%) had an OH < 611. Using a population of 1,945 Japanese Black cattle fathered by 50 sires, Komiya et al. (2021) used a threshold of 288 (1% of SNP) and detected 46 sire–offspring pairs as false, implying a pedigree error rate of 2.4%. Using 5,993 genotyped Limousin cattle, Moore et al. (2019) identified pedigree error rates of between 0.9% for animal and its great-great grandparents and 4% for FS. In comparison, the 0.1% pedigree error rate estimated when using 1% of SNP as the OH threshold highlights the superior quality of pedigree documentation within our dataset. However, it also implies the necessity of incorporating additional metrics alongside OH for more comprehensive paternity testing. Nevertheless, it should be noted that PO relationships are often assessed during the breed society registration process, and this could influence the outcomes of our study, most remarkably the strong agreement between pedigree and genomic information.

On the other extreme, the observed number of OH for unrelated pairs was lower than expected: 4,507.88 vs. 4,600.67 (Table 1). This result was attributed to some kindships not being captured by the pedigree when only 3 generations (i.e., up to grandparents) were sourced. For instance, among our 62,232,954 unrelated pairs, there were 10 pair with an OH ≤ 1,150 (i.e., the expectation for FS pairs) and these had an average additive GR of 0.404 ranging from 0.297 to 0.524 (i.e., well within the ranges for FS pairs or PO pairs).

**Figure 1.**
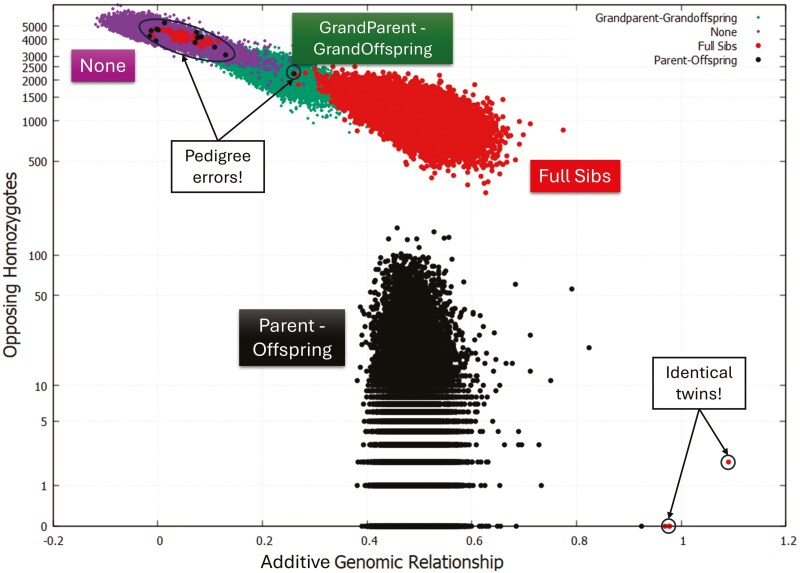
Empirical relationships between additive GR and number of opposing homozygote sites across 4 distinct pedigree-based kinship categories: PO, full sibs, grandparent–grand offspring and none.

Crucially, the anticipated negative correlation between additive GR and OH was affected by the degree of pedigree relationship (Fig. 1) being strongest negative among unrelated pairs (*r* = −0.762 ± 0.001), followed by grandparent–grandoffspring (*r* = −0.740 ± 0.005), HS (*r* = −0.721 ± 0.001), FS (*r* = −0.665 ± 0.004), and PO (*r* = −0.230 ± 0.006) even though their additive GR is like that of FS (Table 1). For 3,540 (or approximately 10%) FS pairs with an observed additive GR > 0.55, the correlation between additive GR and OH was closer to zero at *r* = −0.275 ± 0.016; while for 17,586 (or approximately 82%) PO pairs with 0.45 < additive GR ≤ 0.55 this correlation was merely *r* = −0.028 ± 0.007 indicating the independence between PO additive GR and OH. Also, and consistent with the findings from Fig. S2 showing the presence of 4 FS pairs with a near unity additive GR, Fig. 1 shows these 4 FS pairs with near unity additive GR and OH ≤ 2. These observations reinforce the suspicion that these 4 pairs are, in fact, identical twins.

Regarding the exploration of PO trios, the correlation (± SE) between the observed and the expected additive GR between an offspring and its sire, an offspring and its dam, and an offspring and itself was 0.930 ± 0.003, 0.931 ± 0.004 and 0.689 ± 0.007, respectively. The smaller correlation observed for an offspring and itself was attributed to most of the parents being unrelated with additive GR mean, SD, minimum and maximum of −0.009, 0.043, −0.131, and 0.642, respectively. In fact, in only 13 of the 10,393 trios considered, the additive GR among the parents was > 0.25 (i.e., equivalent of a mating between HS or between grandparent and grandprogeny), and for these 13 instances, the correlation between the observed and the expected additive GR between an offspring and itself was 0.917 ± 0.120.

To close with a broader viewpoint, an exciting feature of this study is its clear demonstration of the value of PCA going beyond cluster detection exercises and into parent-specific measures of prolificacy. At first inspection, the PCA of the additive GR matrix did not reveal the presence of distinct clusters (Fig. S3A). This phenomenon may be attributed to the resource population being made up of a specifically chosen single breed, where the first 2 principal components, PC1 and PC2, together explaining merely 3.25% of the variation in GRs. However, after highlighting the animals based on whether they were parents, it became immediately apparent that parents with lots of progeny were overrepresented in the periphery of the PCA scatter plot, with the most extreme 9 based on PC1 and PC2 having progeny ranging from 145 to 1,221 (arrow pointers in Fig. S3A). Interestingly, when highlighting the progeny and grandprogeny of the 2 most extreme parents based on PC1 and PC2, also having the largest number of progeny, (1,221 and 1,158), we could see clusters of progeny and grandprogeny equidistantly located between the parent and the {0,0} origin (Fig. S3B). The same phenomenon was observed in Fig. S3C highlighting progeny and grand progeny of the next 2 most extreme parents based on PC1 and PC2 with 851 and 486 progeny, respectively. A similar observation, i.e., the progeny of a parent located halfway to the center of the PCA plot, was reported by Mudadu et al. (2016) with 780 Nelore cattle, including 34 prominent sires and their 746 progeny. More recently, with an aim to manage diversity by mating animals across clusters, Steyn et al. (2022) undertook a PCA of an additive GR matrix composed of 1,145 Holstein sires from 6 countries. The authors looked at the animals on the outer edges of the first 12 principal components, resulting in 7 clusters that reduced the total variance by 35%.

Fig. S4 illustrates the network generated through the application of the Pedigromics pipeline (Reverter et al. 2019) to the 10 most prolific sires and dams. These leading parental figures are represented as 20 hubs within the network, labeled S1 to S10 for sires and D1 to D10 for dams. A notable feature of the network is the interconnectivity among 17 of these 20 hubs, with only 3 hubs—D3 from 2011, S8 from 2016, and S9 from 2015—existing in isolation. This interconnectivity is largely sustained by extensive FS families, which are evidently a consequence of the implementation of reproductive biotechnologies. One significant FS family comprises 9 inbred animals (encapsulated by a 7-point star) born in 2021 resulting from the mating of a HS pair, S4 and D5, both of which are descendants of a sire born in 2008 who also sired S3. Additionally, Fig. S4 reveals that D2 and D8 are paternal HS, with D2 serving as the dam of S2, the second most prolific sire.

In conclusion, pedigree relatedness is the expected value of kinship between 2 individuals. The randomness by which a diploid individual transmits alleles to offspring results in actual kinship and inbreeding coefficients varying around the pedigree‐based expected values (Wang, 2016). This randomness can be accurately captured by genome-wide marker data. Goudet et al. (2018) reported discrepancies between pedigree values and marker estimates of kinship and found, via simulations, that these discrepancies were attributable to 2 main sources: pedigree errors and heterogeneity in the origin of founders. Similarly, in our selected population of Australian Angus cattle, both the mean and variance of GRs were close to expectations, while Mendelian inconsistencies were very rare and likely attributed to either errors in pedigree recording, mislabeling of samples, or error in genotypes and genotype imputation. While selecting a population of highly used and genotyped parents along with their progeny also with genotypes is likely to result in the underestimation of pedigree errors, the selective process used to define the resource population was aimed at capturing large and varying types of kindships to be examined. Crucially, this process constitutes a robust method for evaluating inheritance theory and has also allowed us to explore the genetic diversity and breeding management decisions occurring in modern Australian Angus breeding programs. Further potential implications for non-beef cattle scientists and producers can be envisaged particularly where larger full-sib families predominate, carrying larger genomic variation than PO pairs.

## Supplementary Data

Supplementary data are available at *Journal of Animal Science* online.

skaf207_suppl_Supplementary_Tables_S1-S2_Figures_S1-S10

## Abbreviations:

SNP: single nucleotide polymorphism
OH: opposing homozygotes
PO: parent–offspring
FS: full sib
HS: half sib
GR: genomic relationship
PCA: principal component analysis
