# Supplementary material for: Short communication: genomic kinship, opposing homozygotes and genetic diversity in a selected population of Australian Angus cattle
Source: J Anim Sci. 2025 Jun 19;103:skaf207. doi: 10.1093/jas/skaf207 (PMC12267139; doi:10.1093/jas/skaf207)
Supplement: skaf207_suppl_Supplementary_Tables_S1-S2_Figures_S1-S10 [file skaf207_suppl_supplementary_tables_s1-s2_figures_s1-s10.pdf]

SUPPLEMENTARY MATERIAL TO

Short Communication: Genomic kinship, opposing homozygotes and genetic diversity in a selected population of Australian Angus cattle[1]

Antonio Reverter[*,2], Malshani Samaraweera[†], Pâmela A. Alexandre[*], Christian Duff[†] and Laercio Porto-Neto[*]

[*]CSIRO Agriculture and Food, 306 Carmody Rd., St. Lucia, Brisbane, Qld 4067, Australia.

[†]Angus Australia, 86 Glen Innes Road, Armidale, NSW 2350, Australia.

[2]Corresponding author: Toni.Reverter-Gomez@csiro.au

FIGURES AND TABLES

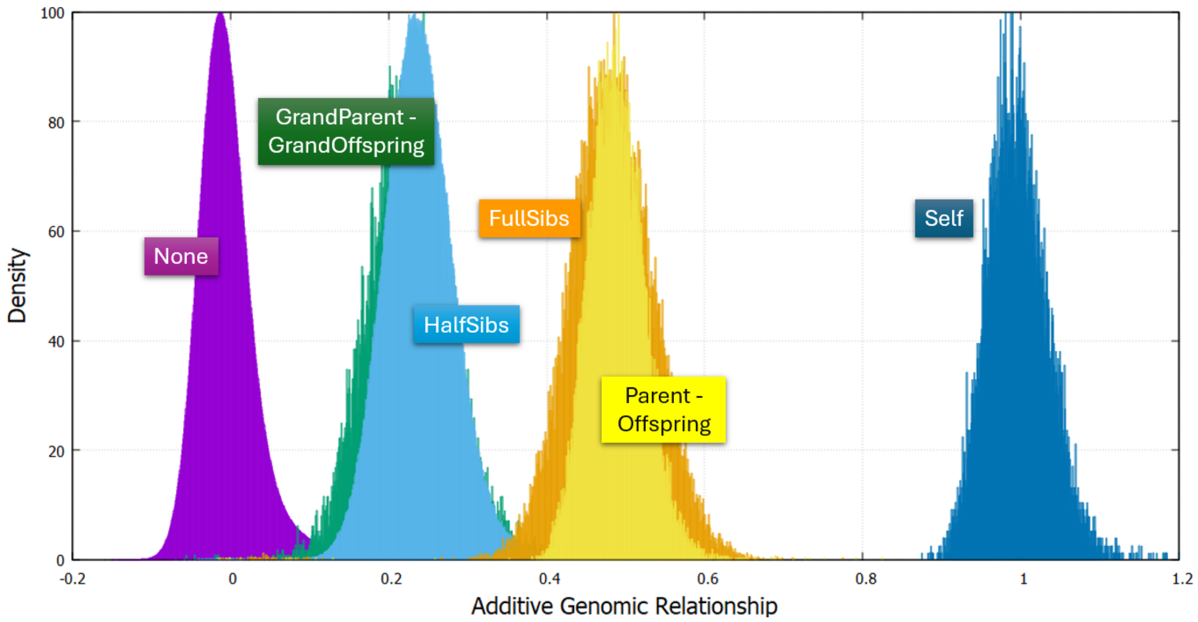

Supplementary Figure 1. Empirical density distribution for additive genomic relationships across six distinct pedigree-based kinship categories: Self relationships, parent-offspring, full sibs, half sibs, grandparent – grand offspring and none.

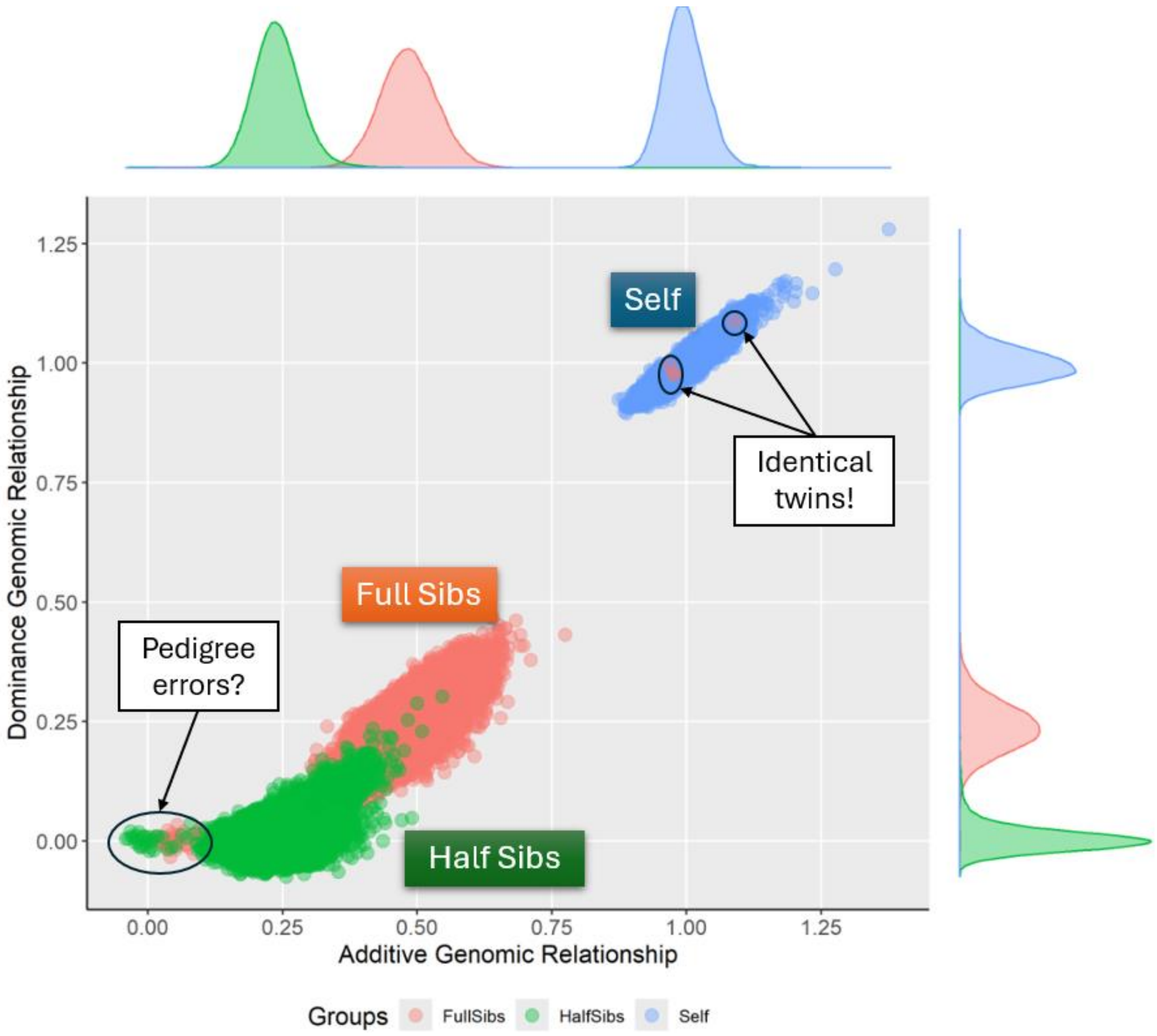

Supplementary Figure 2. Empirical relationships between additive and dominance genomic relationship across three distinct pedigree-based kinship categories: Self relationships, full sibs and half sibs.

(A)

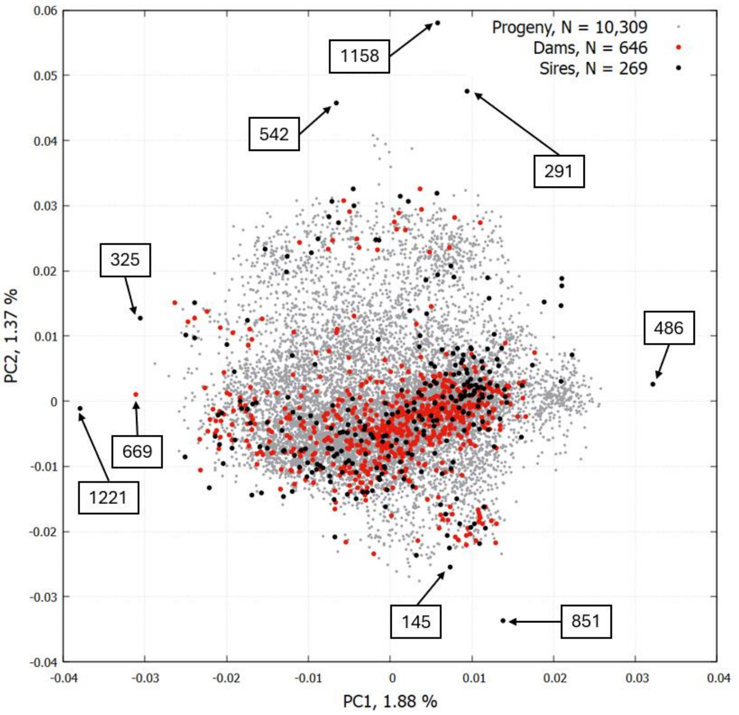

(B) **(C)**

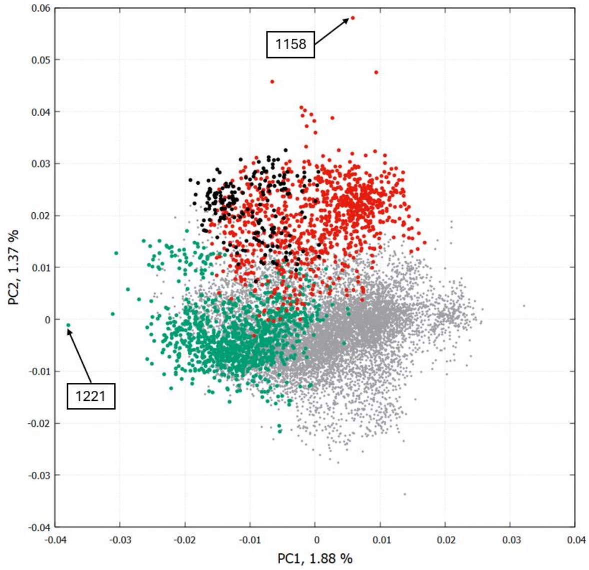

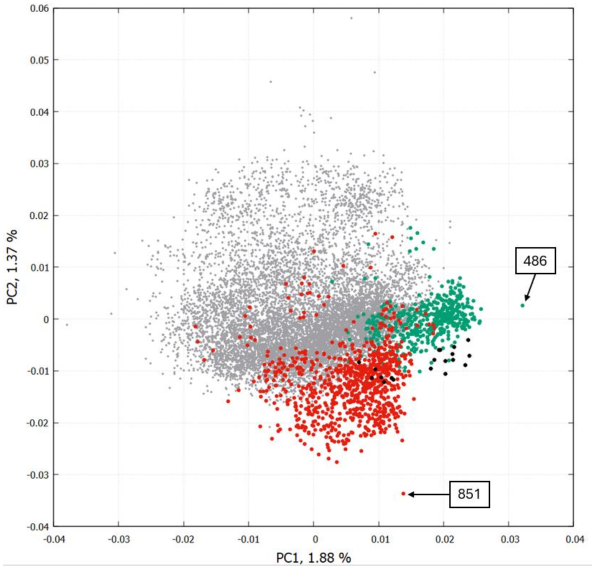

Supplementary Figure 3. The first two principal components, PC1 and PC2, based on the additive genomic relationship matrix of 11,224 animals including 10,309 progeny, 646 dams and 269 sires: (A) Highlighting progeny, dams and sires and number of progeny of parents exhibiting extreme values for either PC1 or PC2; (B) Highlighting progeny and grand progeny of two sires with 1,221 (green) and 1,158 progeny (red) and common progeny or grand progeny (black); (C) Highlighting progeny and grand progeny of two sires with 851 (red) and 486 progeny (green) and common progeny or grand progeny (black).

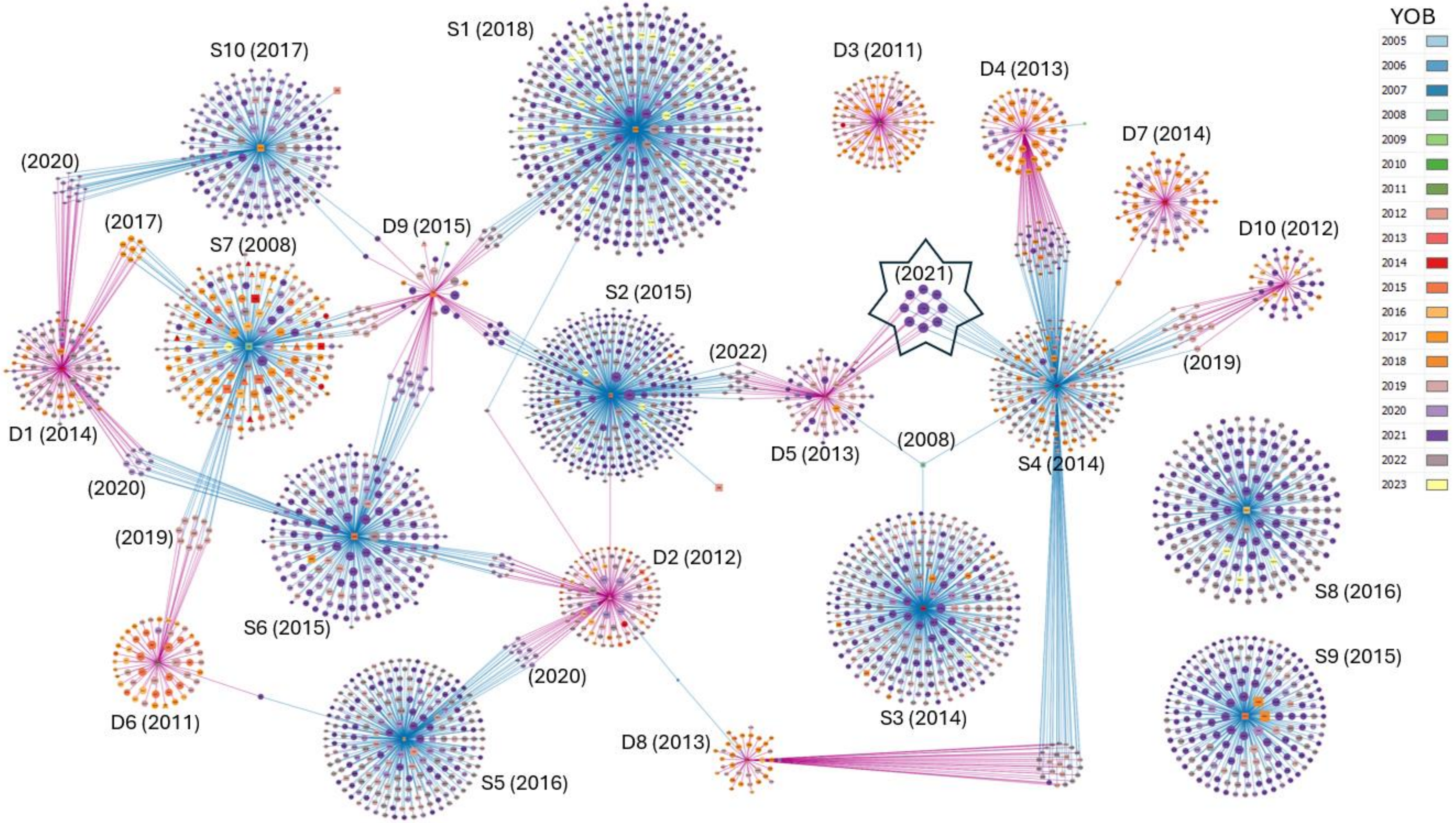

Supplementary Figure 4. Pedigromics view of the interconnectivity existing among the ten most prolific sires and dams, labelled S1 to S10 for sires and D1 to D10 for dams. Year of birth are mapped to colors and given in bracket for selected individuals. Blue and red edges represent sire-offspring and dam-offspring relationships, respectively. Node size reflects the level of inbreeding.

Supplementary Table 1. Summary statistics (expected (Exp), mean, SD, minimum and maximum) for additive and dominance genomic relationship (GR) across ten pedigree-based relationships examined in this study.

Relationship	N Pairs	Additive GR					Dominance GR				
		Exp.	Mean	SD	Min.	Max.	Exp.	Mean	SD	Min.	Max.
Self	11,224	1.000	0.995	0.038	0.874	1.376	1.000	0.997	0.036	0.894	1.279
Full sib	35,486	0.500	0.483	0.054	-0.009	1.090	0.250	0.239	0.052	-0.033	1.086
Sire-Offspring	10,892	0.500	0.485	0.036	-0.015	0.824	0.000	-0.004	0.030	-0.106	0.428
Dam-Offspring	10,415	0.500	0.492	0.037	-0.004	0.923	0.000	-0.004	0.030	-0.097	0.336
Paternal half sib	584,845	0.250	0.239	0.044	-0.069	0.584	0.000	0.005	0.027	-0.088	0.354
Maternal half sib	92,576	0.250	0.240	0.048	-0.059	0.543	0.000	0.002	0.030	-0.094	0.253
Paternal grandparent	8,059	0.250	0.225	0.050	-0.044	0.437	0.000	-0.003	0.021	-0.073	0.114
Maternal grandparent	8,215	0.250	0.228	0.053	-0.056	0.480	0.000	-0.005	0.022	-0.080	0.153
Paternal and maternal grandparent	34	0.500	0.469	0.066	0.346	0.642	0.125	0.109	0.057	0.020	0.297
None	62,232,954	0.000	-0.003	0.038	-0.158	0.533	0.000	0.000	0.012	-0.091	0.216

Supplementary Table 2. Summary statistics (mean, SD, minimum and maximum) for expected and observed number of opposing homozygotes (OH) sites across ten pedigree-based relationships examined in this study.

Relationship	N Pairs	Expected OH		Observed OH			
		Equation	N	Mean	SD	Min.	Max.
Self	11,224	0	0	0	0	0	0
Full sib	35,486	$\sum ½\, p^2q^2$	1,150.57	1,162.45	286.811	0	4,698
Sire-Offspring	10,892	0	0	7.61	89.555	0	4,662
Dam-Offspring	10,415	0	0	15.74	122.403	0	5,290
Paternal half sib	584,845	$\sum p^2q^2$	2,300.34	2,268.40	352.754	642	5,744
Maternal half sib	92,576	$\sum p^2q^2$	2,300.34	2,262.03	362.211	18	5,224
Paternal grandparent	8,059	$\sum p^2q^2$	2,300.34	2,316.99	417.580	895	4,537
Maternal grandparent	8,215	$\sum p^2q^2$	2,300.34	2,298.27	419.669	820	4,740
Paternal and maternal grandparent	34	$\sum ½\, p^2q^2$	1,150.57	1,245.68	476.408	496	3,050
None	62,232,954	$\sum 2p^2q^2$	4,600.67	4,507.88	449.325	2	9,512
